# BED domain‐containing NLR from wild barley confers resistance to leaf rust

**DOI:** 10.1111/pbi.13542

**Published:** 2021-03-06

**Authors:** Chunhong Chen, Matthias Jost, Bethany Clark, Matthew Martin, Oadi Matny, Brian J. Steffenson, Jerome D. Franckowiak, Martin Mascher, Davinder Singh, Dragan Perovic, Terese Richardson, Sambasivam Periyannan, Evans S. Lagudah, Robert F. Park, Peter M. Dracatos

**Affiliations:** ^1^ Agriculture & Food Commonwealth Scientific and Industrial Research Organisation Canberra ACT Australia; ^2^ Plant Breeding Institute The University of Sydney Cobbitty NSW Australia; ^3^ Department of Plant Pathology University of Minnesota St. Paul MN USA; ^4^ Department of Agronomy and Plant Science University of Minnesota St. Paul MN USA; ^5^ Leibniz Institute of Plant Genetics and Crop Plant Research (IPK) Gatersleben Seeland Germany; ^6^ German Centre for Integrative Biodiversity Research (iDiv) Halle‐Jena‐Leipzig Leipzig Germany; ^7^ Institute for Resistance Research and Stress Tolerance Federal Research Centre for Cultivated Plants Julius Kühn‐Institute (JKI) Quedlinburg Germany

**Keywords:** Gene cloning, leaf rust resistance, NLR, wild barley

## Abstract

Leaf rust, caused by *Puccinia hordei,* is a devastating fungal disease affecting barley (*Hordeum vulgare* subsp. *vulgare*) production globally. Despite the effectiveness of genetic resistance, the deployment of single genes often compromises durability due to the emergence of virulent *P. hordei* races, prompting the search for new sources of resistance. Here we report on the cloning of *Rph15*, a resistance gene derived from barley’s wild progenitor *H. vulgare* subsp. *spontaneum*. We demonstrate using introgression mapping, mutation and complementation that the *Rph15* gene from the near‐isogenic line (NIL) Bowman + *Rph15* (referred to as BW719) encodes a coiled‐coil nucleotide‐binding leucine‐rich repeat (NLR) protein with an integrated Zinc finger BED (ZF‐BED) domain. A predicted KASP marker was developed and validated across a collection of Australian cultivars and a series of introgression lines in the Bowman background known to carry the *Rph15* resistance. *Rph16* from HS‐680, another wild barley derived leaf rust resistance gene, was previously mapped to the same genomic region on chromosome 2H and was assumed to be allelic with *Rph15* based on genetic studies. Both sequence analysis, race specificity and the identification of a knockout mutant in the HS‐680 background suggest that *Rph15‐* and *Rph16*‐mediated resistances are in fact the same and not allelic as previously thought. The cloning of *Rph15* now permits efficient gene deployment and the production of resistance gene cassettes for sustained leaf rust control.

## Introduction

In 2018, barley (*Hordeum vulgare* subsp. *vulgare*) was ranked fourth amongst grain crops in production (141 million tonnes) behind maize, rice and wheat (FAOSTAT, 2018). It is used primarily for animal feed and malt production, but also serves as a major food staple in the mountainous areas of Central Asia, Southwest Asia, the Andes of South America and North Africa. Leaf rust, caused by the fungus *Puccinia hordei* Otth, is the most damaging and widespread rust disease of barley (Park *et al*., 2015). Leaf rust epidemics can occur in most if not all barley growing regions and have been reported to cause significant reductions in grain quality and yield. Yield losses up to 62% have been reported in highly susceptible barley cultivars (Cotterill *et al*., 1992). Resistance to leaf rust in barley is conferred by either qualitative type *Rph* (Reaction to *Puccinia hordei*), all‐stage resistances (ASR) that are typically race‐specific or by quantitative trait loci (QTL) conferring partial adult plant resistance (APR) that is generally not race‐specific (Niks *et al*., 2015). Due to *P. hordei* evolution and the subsequent emergence of new pathogenic variants, ASR resistance to leaf rust is often transiently effective. Of the 22 catalogued ASR genes, five (*Rph10*, *Rph11*, *Rph13*, *Rph15* and *Rph16*) originate from *H*. *v*. ssp. *spontaneum* (Park *et al*., 2015).

**Figure 1:**
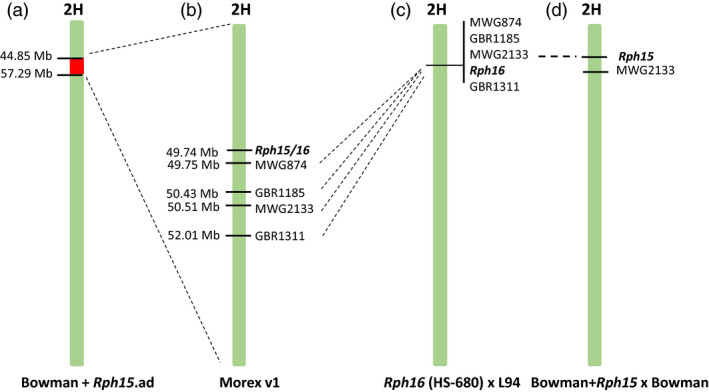
Comparative physical and genetic maps for chromosome 2H at the *Rph15* locus, including graphical genotypes for (a) Bowman introgression line BW 719 carrying leaf rust resistance gene *Rph15* from Martin *et al*. (2020a) and (b) the corresponding physical region in the Morex V1 reference genome assembly used to search for candidate NLR genes for further functional analysis (Mascher *et al*., 2017). The red coloured section within the BW719 chromosome 2H represents the 12.5Mb region of retained donor chromatin from PI 355447 (*Rph15*). Previously published genetic maps for chromosome 2H derived from mapping populations including: (c) HS‐680 × L94 (Perovic *et al*., 2004) and (d) Bowman + *Rph15* × Bowman (Weerasena *et al*., 2004) used to map *Rph16* and *Rph15*, respectively, were included and cosegregating RFLP markers were anchored onto the Morex physical assembly.

Leaf rust resistance gene *Rph15* was originally sourced from PI 355447, an accession of wild barley collected from Israel (Chicaiza, 1996; Jin and Steffenson, 1994). The reported widespread effectiveness of the *Rph15* resistance to >350 *P. hordei* isolates likely reflects its limited deployment in agriculture (Martin *et al*., 2020a). Virulence for *Rph15* does, however, exist in nature and was identified in an Israeli isolate (90‐3) from a global survey, presaging the possible breakdown of *Rph15* should it be deployed singly in cultivars (Martin *et al*., 2020a). Given the apparent rare occurrence of virulence for *Rph15*, it represents a valuable gene for leaf rust control in cultivated barley when deployed in combination with other effective resistance genes especially given the limited number of *Rph* genes that have been isolated to date. Chicaiza (1996) first determined that the *Rph15* resistance was inherited as a single dominant gene that mapped to the centromeric region of the short arm of chromosome 2H in close linkage to the RFLP marker MWG2133. Martin *et al*. (2020a) recently developed a series of introgression lines (*Rph1‐Rph15*) in the genetic background of cultivar Bowman and mapped *Rph15* to a physical interval (44‐57Mb) on chromosome 2H in the Morex reference genome (Figure 1). Genetic mapping of resistance genes *Rph15* and *Rph16* on chromosome 2H was originally performed using the Bowman + *Rph15* × Bowman (Weerasena *et al*., 2004) and the HS‐680 × L94 (Perovic *et al*., 2004) mapping populations, respectively. Further genetic studies determined that *Rph15* and *Rph16* from wild barley were likely allelic, and both were closely linked or colocated with RFLP marker MWG2133 (Weerasena *et al*., 2004). Other studies have repeatedly mapped leaf rust resistance at the *Rph15* locus*,* suggesting the possible presence of an allelic series or complex resistance locus on chromosome 2H (Derevnina *et al*., 2014; Ivandic *et al*., 1998; Perovic *et al*., 2004; Weerasena *et al*., 2004). In this study, our aim was to isolate the *Rph15* resistance gene and assess the molecular basis of the postulated allelic relationship between *Rph15* and *Rph16* on the short arm of chromosome 2H.

## Results and discussion

The approach used in this study for the cloning of *Rph15* was based on two hypotheses. The first hypothesis was that based on the susceptibility of cultivar Morex to races of *P. hordei* that are avirulent for *Rph15*, its sequenced genome (Mascher *et al*., 2017) may be useful for identifying the *rph15* susceptibility allele within the physical interval reported by Martin *et al*. (2020a) (Figure 1). The second hypothesis was that due to the characteristic hypersensitive response associated with *Rph15*‐mediated resistance, we anticipated the involvement of immune receptors encoded by R genes such as nucleotide‐binding site leucine‐rich repeats (NLRs). The 12.5 Mb introgressed segment defined for the near‐isogenic line (NIL) Bowman + *Rph15* (referred to as BW719) was analysed for the presence of NLR genes using the NLR annotator tool, which is an automatic high‐throughput analysis for the NLR‐specific conserved motif combinations (Steuernagel *et al*., 2020). A single NLR was identified represented by the gene model HORVU2Hr1G019120.5 from the Morex reference (Mascher *et al*., 2017) and was deemed the best candidate for further investigations based on a loss of function mutant population derived from the BW719 background.

**Figure 2:**
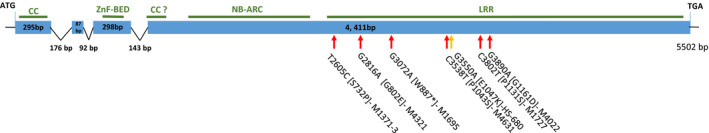
Model of the *Rph15* gene amplified from BW719 near‐isogenic line using primers designed to candidate gene HORVU2Hr1G019120.5. The gene consists of four exons (blue boxes) and three introns (black lines). The CC and NB‐ARC domains were predicted using the NLR annotator software developed by Steuernagel *et al.,* (2020) and the LRR motifs were predicted as described by Martin *et al.,* (2020b). Red arrows indicate the positions of susceptible mutants identified within the BW719 (*Rph15*) background and the orange arrow was a single mutant identified in the HS‐680 (*Rph16*) background. Areas spanning conserved domains are highlighted in green.

A mutagenesis‐based approach was used to verify the HORVU2Hr1G019120.5 candidate as the gene of interest and chemically treated 1500 seed sourced from the same BW719 stocks reported in Martin *et al*. (2020a). To confirm the BW719 stock was the same as that used to map the introgression in Martin *et al*. (2020a) to use for mutagenesis, we reconfirmed the previously reported monogenic inheritance and linkage of *Rph15* with RFLP marker MWG2133 on chromosome 2H using a Bowman + *Rph15* × Gus F_2:3_ population (Figure S1). Rust testing of progeny seeds from 4320 M_2_ spikes with the *Rph15*‐avirulent *P. hordei* isolate 5457 Pj + identified eight independent mutants (*rph15*) that were confirmed at the M_3_ generation. A 9442 base pair (bp) full‐length genomic sequence that included 1.3 kb and 2.5 kb of 5′ and 3′ sequence, respectively, was amplified from Bowman + *Rph15* using primers designed to the candidate gene model (HORVU2Hr1G019120.5) based on the Morex (*rph15*) sequence. The NLR motif prediction was used to develop a full‐length NLR candidate gene model annotation (Figure 2). We then sequenced the full‐length gene from the eight mutants and identified non‐synonymous mutations in six out of the eight lines including: a premature stop codon (M1695), serine to proline substitutions or *vice versa* (M1371‐3, M1727 and M4022) and substitutions from glycine to either glutamic or aspartic acid (M4022 and M4321). All non‐synonymous mutations were identified in the LRR region of the *Rph15* gene (Figure 2; Figure S2). The remaining two susceptible mutants did not contain sequence changes in the candidate gene, suggesting they likely carry non‐synonymous mutations within downstream signalling components essential for *Rph15*‐mediated resistance. Similar findings were also identified during the recent cloning of the leaf rust resistance gene *Rph1* from cultivated barley (Dracatos *et al*., 2019).

A KASP marker was developed by interrogating a G/C SNP within the 3rd intron of the *Rph15* gene that was further validated as predictive for the *Rph15* resistance. We assessed the utility of the KASP marker developed within the *Rph15* gene across 61 near‐isogenic lines in the Bowman background (BW lines – Martin *et al*., 2020a) and 80 Australian cultivars that lack the *Rph15* resistance based on extensive leaf rust phenotyping. All 80 Australian cultivars lacked the *Rph15* marker allele corroborating their phenotypic response. Based on data reported in Martin *et al*. (2020a), from the 61 BW lines tested for rust resistance, 50 were postulated to carry *Rph15* either singly or in combination with another resistance gene based on the presence of the *Rph15* KASP marker allele, whilst the remaining 11 were postulated to carry unknown resistances (Table S1). Interestingly, six of the BW lines postulated to carry *Rph15* and a further six BW lines with unknown leaf rust resistance failed to amplify (same signal profile as water blank) and likely carry a SNP haplotype at the marker different to either Bowman or BW719. Further work is underway to determine whether these BW lines carry uncharacterized resistance or allelic variants of the *Rph15* gene (Table S1).

**Figure 3:**
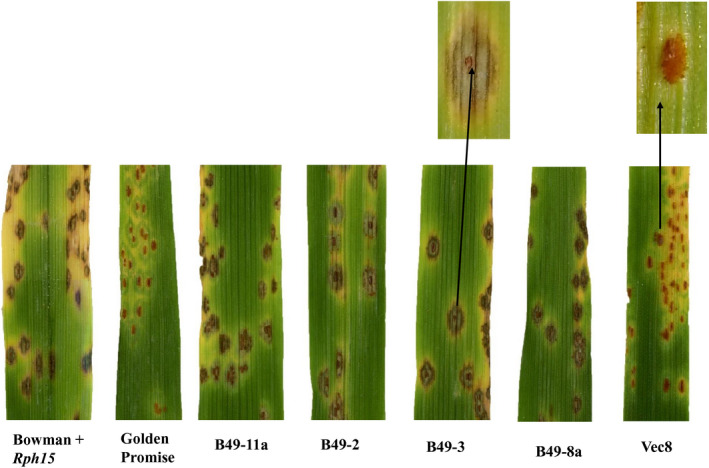
Complementation of the wild‐type *Rph15* gene from BW719 into Golden Promise using Agrobacterium‐mediated transformation. Resistant sib plants from four different Golden Promise + *Rph15* T_1_ generation families showed the same resistance reaction in response to the *Rph15*‐avirulent *Puccinia hordei* race 5457 P + 12 days after infection as the Bowman + *Rph15* control relative to the susceptible cv. Golden Promise and the empty vector control Vec8.

The full‐length genomic sequence of *Rph15* from BW719 was cloned into a binary vector and transformed into the leaf rust susceptible barley cultivar Golden Promise. Six independent hemizygous T_0_ lines were produced that contained the wild‐type *Rph15* candidate in addition to two control lines that contained an empty vector only (Figure S3). The progeny from four T_1_ Golden Promise + *Rph15* transgenic families was evaluated at the seedling stage in Australia with *Rph15*‐avirulent pathotype 5457 P+ and in North America with the *Rph15*‐avirulent *P. hordei* pathotype 92‐7 and the only known *Rph15*‐virulent *P. hordei* isolate 90‐3 collected from Israel (Figure S4; Table S2; Table S3). All four Golden Promise + *Rph15* T_1_ generation transgenic lines showed the expected segregation for low infection types (0;CN‐;1CN) characteristic of the *Rph15* resistance observed on BW719 seedlings in response to both *Rph15*‐avirulent *P. hordei* isolates (Figure 3). In response to the *Rph15*‐virulent isolate (90‐3), nearly all plants assessed from the four transgenic T_1_ families were susceptible; however, variation was observed between the two experimental replicates due to the likely presence of inoculation escapes in replicate 1 [evidenced by the presence of a few plants that were immune (00;) with no necrosis that was uncharacteristic of the *Rph15* resistance] (Table S3). Based on qRT‐PCR data, the relative expression of *Rph15* was variable both within and between T_1_ families possibly reflecting differences in copy number or the site of T‐DNA integration in the genome of Golden Promise and the assessment of siblings that were either heterozygous or homozygous for the *Rph15* transgene/s (Figure S5).

To confirm the correlation between the presence of the transgene and the observed phenotypic response in the T_1_ transgenic families, 12 sib plants per T_1_ family were genotyped with the predictive KASP marker. Genotypic analysis confirmed, without exception, cosegregation between the presence of the heterozygous SNP genotype (Golden Promise susceptibility allele in combination with the T‐DNA from BW719 in all resistant sibs, and only the susceptibility allele found within all susceptible sib lines). Thus, the exploitation of previous mapping data, combined with both mutation and complementation experiments in the present study, facilitated confirmation of the candidate as the causal gene for *Rph15‐*mediated resistance.

**Figure 4:**
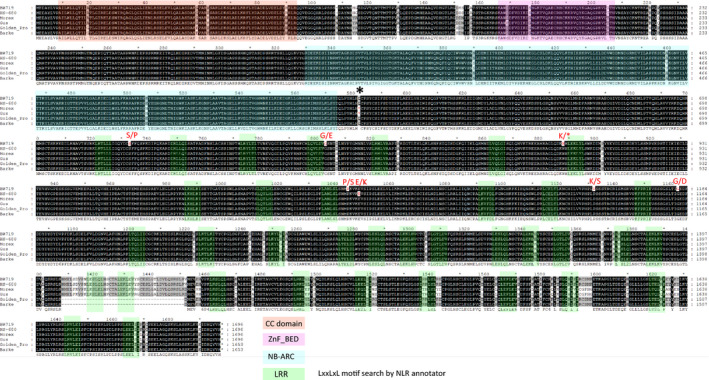
Protein alignment between BW719 (*Rph15*) and HS‐680 (*Rph16*) and a set of susceptible cultivars Morex, Barke, Golden Promise and Gus of the encoded Rph15 amino acid sequence. The protein sequences were highly conserved, the alignment indicated the presence of two distinct susceptibility haplotypes relative to the Rph15 resistance haplotype. The detected conserved domains were identified using the NLR annotator described by Steuernagel et al. (2020) as well as the reanalysis of the LRR motif using the LxxLxL motif search, Martin et al., (2020b) are highlighted in colours according the legend. All detected mutants (highlighted in red) are located in the LRR region of the protein. The critical SNP haplotype characterized by a Glutamine to a premature STOP codon is denoted by a bold asterix.

Rare sub‐families of NLR immune receptors in plants have evolved to encode additional integrated domains that act as decoys to recognize and interact with pathogen effectors that would normally target host transcription factors containing zinc finger BED (ZF‐BED) domains (Grund *et al*., 2019; Kroj *et al*., 2016). The ZF‐BED domain was originally characterized in *Drosophila* through mutagenesis studies and was subsequently shown to be essential in rice for *Xa1*‐mediated resistance to bacterial blight (Yoshimura *et al*., 1998). More recently, three BED domain NLRs (*Yr5*, *Yr7* and *YrSP* on the long arm of chromosome 2B) with unique pathogen specificity conferred resistance to stripe rust in bread wheat, comprising a complex resistance NLR cluster (Marchal *et al*., 2018). Sequencing of the cDNA from BW719 indicated that the *Rph15* gene consists of four (three small and one large) exons and encodes an immune receptor with a single predicted BED domain followed by the canonical NLR (1696 residues) (Figures 2 and 4). The presence of four exons and only a single BED domain differs from the three exons structure identified for BED domain‐containing NLRs (*Yr5/Yr7/YrSP*) in bread wheat (Marchal *et al*., 2018). The identification of a BED domain within the encoded Rph15 immune receptor reveals that different rust pathogen species adapted to distinct hosts may have effectors targeting similar transcription factor domains.

**Figure 5:**
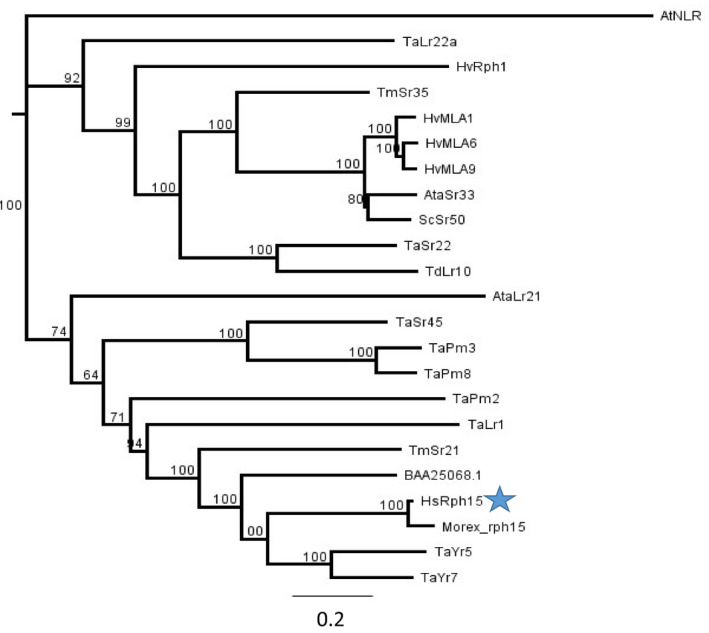
Phylogenetic analysis was performed by multiple alignment of Rph15 from BW719 with full‐length cloned NLRs from the Triticeae and was visualized using a Neighbour‐joining tree analysis. An alignment was performed both with and without the BED domain and no noticeable differences in node length or clustering was identified. Rph15 (denoted with star) clustered with other BED domain‐containing NLRs from wheat and rice and was more distantly related to Rph1 from cultivated barley and the MLA clade. Statistical support for individual nodes were estimated from 1000 bootstrap replicates and values are represented as percentages on the nodes (values of. 70% are shown). The scale bar represented the proportion of site changes along each branch.

Phylogenetic analysis using numerous recently cloned NLR immune receptors from the Triticeae determined that Rph15 was most closely related to the susceptible version of the Rph15 protein from Morex followed by other BED domain carrying NLR proteins, including both Yr5 and Yr7 homologues from wheat and Xa1 from rice on chromosome 4 (corresponding to 2H in barley) (Figure 5). Interestingly, the phylogenetic relatedness between NLRs was not based on the presence or absence of a BED domain as we performed sequence alignments using full‐length NLRs both with and without the BED domains (Figure 5). Rph15 was closely related to the stem rust resistance protein sequence of Sr21 from *Triticum monococcum* and other NLRs conferring rust and mildew resistance from bread wheat and its relatives whilst the MLA clade and the recently cloned leaf rust resistance gene encoding Rph1 from cultivated barley clustered separately from Rph15 (Dracatos *et al*., 2019; Figure 5). Further examination of the BED domains between Rph15*,* Yr5/Yr7 and Xa1 suggests that the BED domain from Rph15 was more closely related to the BED II clade consensus and yet only carried six out of the nine conserved residues, suggesting the possible presence of a functionally diverse BED domain distinct from BED I and II (Figure S6). According to Marchal *et al*. (2018), Yr5 and Yr7 carried BED domains that clustered with other clade I BED domain carrying NLRs from the Triticeae, whilst Xa1 from rice clustered separately from both clades I and II yet still carried eight out of the nine conserved residues between BED I and II clades. Further experiments assessing both the function and involvement of the BED domain in the *Rph15*‐mediated resistance are required to test this hypothesis.

To determine the functional SNP variant/s required for *Rph15*‐mediated resistance, we aligned Rph15 protein sequence with homologues from four different cultivated barley genotypes that lack the *Rph15* resistance, *viz*. Morex, Barke, Gus and Golden Promise (Mascher *et al*., 2017; Schreiber et al. 2020). Despite all barley genotypes assessed having distinct haplotypes, three main diverse haplogroups were identified amongst the six sequences: the *Rph15* resistance haplotype and two distinct susceptibility haplotypes *rph15_1* and *rph15_2* (Figure 4). The Gus and Morex rph15 proteins differed by a single amino acid substitution, and their *rph15_1* haplotype was characterized by numerous non‐synonymous SNPs mainly in the LRR domain. One critical SNP was identified that caused a substitution from a cysteine to a premature STOP codon (Figure 4). The other susceptible haplotype *rph15_2* was identified in both Barke and Golden Promise and differed by >45 residues largely due to a deletion event of 30 residues relative to the other haplotypes also in the LRR domain. Based on the alignment, we resolved the difference between resistance and susceptibility to six functional SNP variants. Further sequencing efforts of additional barley accessions is likely to determine how many susceptibility haplotypes exist within the *Hordeum* gene pool and permit the identification of the causal polymorphisms differentiating susceptibility vs *Rph15*‐mediated resistance. Many of these variants can be further interrogated for diagnostic marker design and future gene‐editing applications.

The discovery of two other widely resistant *H. vulgare* sp. *spontaneum* accessions (HS078 and HS‐680) led to the identification of another leaf rust resistance allele at the *Rph15* locus (Ivandic *et al*., 1998). The development of segregating doubled haploid (DH) populations using the susceptible line, L94 and subsequent genetic map construction led to the mapping of the resistance loci of both HS078 and HS‐680 in the short arm of chromosome 2H via linkage with RFLP markers MWG784 and MWG2133 (Ivandic *et al*., 1998). An early study previously mapped *Rph15* to the long arm of chromosome 2H; therefore, the gene in both HS078 and HS‐680 was predicted as independent and was catalogued as *Rph16* (Ivandic *et al*., 1998). Weerasena *et al*. (2004) subsequently concluded that based on the lack of segregation observed in 1027 F_2_ individuals from the cross BW719 × HS‐680, *Rph15* and *Rph16* are likely alleles of each other. Furthermore, closely linked AFLP markers led to a change in map position from the long arm to the short arm of chromosome 2H. Hence *Rph15* was in close proximity to *Rph16* based on cosegregation with RFLP marker MGW2133. To further unravel the genetic relationships between *Rph15* and *Rph16*, in this study, we used the same primer pairs used to PCR‐amplify the *Rph15* gene from BW719 to determine the *Rph15* haplotype in HS‐680. Both BW719 and HS‐680 had identical (100% nucleotide identity over 9.5kb) *Rph15* haplotypes; therefore, we propose an alternative hypothesis, that the *Rph16* resistance in HS‐680 is due to the same BED domain‐containing NLR conferring *Rph15*‐mediated resistance originally sourced from PI 355447 in BW719.

Examination of historical phenotypic data using five *Rph15*‐avirulent isolates from diverse origins and one *Rph15*‐virulent isolate from Israel determined that *Rph15* and *Rph16* shared the same race specificity (Table S4). Furthermore, phenotypic screening of an EMS HS‐680 mutant population using an avirulent *P. hordei* isolate (I‐80) and subsequent Sanger sequencing identified a single non‐synonymous G to A knockout *rph15* mutation in two sib lines (Figure 2; Figure 4). Due to chance the possible involvement of second neighbouring gene also conferring *Rph16*‐mediated resistance cannot be ruled out, however further comparative genomic analysis based on sequencing of knockout and/or the HS‐680 wild type is required to test the two‐gene hypothesis.

The level of SNP marker diversity near the *Rph15* allele is similar to that observed with barley genes that originated before the domestication of barley. Martin *et al*. (2020a) reported on the development and characterization of numerous *Rph* introgression lines in cultivar Bowman (BW lines). With 20 different haplotype patterns in the 50 BW lines likely to have the *Rph15* resistance allele, the marker diversity near the *Rph15* allele is greater than that observed for mutant genes that involved during or after domestication of barley such as non‐brittle rachis (*btr1* and *btr2*) (Casas *et al*., 2018) and six‐rowed spike type (*vrs1*) (Pourkheirandish *et al*., 2015).

Three BW lines with the *Rph15* resistance allele have the same T2H‐4H reciprocal translocation with the breakpoint in 2HS distal to the *Rph15* locus. The three BW lines (BW723 from PI 405179, BW724 from PI 405341 and BW725 from PI 391004) have identical marker haplotypes in the interstitial regions between the breakpoint and the centromere. This T2H‐4H translocation is different from the T2H‐4H translocation in *H. v*. subsp. *spontaneum* described by Konishi and Linde‐Laursen (1988) because the wild barley accessions originated from Israel instead of Russia and the breakpoint is in a sub‐terminal position of 2HS, at about 50.5 Mb (53.3 cM), instead of near the centromere. Because survival of gametes with crossovers in the interstitial regions is extremely rare and there is no evidence that the critical allele for *Rph15* originated more than once, *Rph15* must have been present in *H. v*. subsp. *spontaneum* before the translocation event occurred. These observations suggest that the *Rph15* resistance allele evolved prior to the domestication of barley. The emergence of the *rph15_1* haplotype defined by the premature STOP codon variant may also explain the loss of *Rph15*‐mediated resistance in the cultivated barley gene pool.

The wild barley *Rph15* donor accession PI 355447 was originally collected in Israel and confers resistance to a wide range of *P. hordei* isolates (Chicaiza, 1996; Weerasena *et al*., 2004). We examined exome capture data developed from a previously characterized panel of 267 accessions based on the 2017 Morex assembly comprised of both wild and cultivated barley genotypes (Mascher *et al*., 2017; Russell *et al*., 2016). Due to the featured premature STOP codon in Morex (*rph15_1*), only a truncated *rph15* sequence was available for comparison. Extensive functional diversity was identified amongst the 267 barleys; however, no wild accession carried the *Rph15* allele sequence. Five nearest neighbours with 80‐90% nucleotide similarity were identified in the Western Fertile Crescent region bordering Israel and Jordan (Figure S7); however, further detailed SNP haplotyping is required to determine the precise origin and distribution of the *Rph15* resistance.

A single virulent *P. hordei* isolate was collected in close geographic proximity to the predicted origin of *Rph15*. We superimposed the location of the collection site of isolate 90‐3 and determined it colocated with the putative origin of *Rph15* near Tel Aviv (Figure S7). This highlights the ability of surrounding *P. hordei* pathogen populations to acquire virulence and overcome a resistance gene allele present in wild barley populations in Israel. Furthermore, the presence of the alternate host (star‐of‐Bethlehem – *Ornithogalum umbellatum*) in this region also may have been a contributing factor for the emergence of virulence for *Rph15*. Despite its wide effectiveness, to date *Rph15* has only been deployed in one barley cultivar (ND Genesis, PI 677345) in the Upper Midwest region of the USA, and its durability in agriculture remains unknown. Although NLR resistance genes have often been overcome by matching pathogen virulence, there is evidence that they can be an important component of durable resistance when deployed in combination with resistance genes of different structure and function (Palloix *et al*., 2009).

Given that the number of leaf rust resistance genes in barley is finite, it is crucial to use these genes with appropriate stewardship to ensure they remain effective for as long as possible. Experience has shown that the most effective strategy to protect resistance genes and achieve durable disease resistance in crop species such as wheat (Park, 2007) and barley (Park *et al*., 2015) is to deploy multiple genes in combination. Depending on public acceptance, *Rph15* could also be pyramided in resistance gene cassettes in combination with other cloned resistance gene alleles such as *Rphq2* (Wang *et al*., 2019) and *Rph1* (Dracatos *et al*., 2019) using a cisgenic approach. Effective gene deployment is reliant on selection of suitable gene combinations and the presence of diagnostic molecular tools to reduce the possibility of hitchhiking effects of deleterious traits. From a breeding perspective, we propose a strategy of combining *Rph15* with *Rph20*, a gene conferring partial adult plant resistance (*Rphq4* in Qi *et al*., 1998). These genes are not located near any loci controlling undesirable traits, as evidenced by the deployment of *Rph15* in ND Genesis and the widespread use of *Rph20* for over 70 years during which time it has remained durable. Although not yet cloned, the mode of action of *Rph20* likely differs from *Rph15* based on previously reported mechanisms of adult plant resistances derived from bread wheat (Krattinger *et al*., 2009; Moore *et al*., 2015). Therefore, to overcome this gene combination, the pathogen would need at least two mutations (either sequential or simultaneous) in contrasting response mechanisms.

In summary, we have cloned the leaf rust resistance gene *Rph15* originating from wild barley accession PI 355447 collected in Israel and determined it encodes a BED domain‐containing immune receptor. Sequence comparison between wild type and mutant in the donor accession for *Rph16* (HS‐680) suggests that *Rph15* and *Rph16* are neither allelic nor distinct closely linked genes as previously thought but in fact the same gene; however, the involvement of second neighbouring gene cannot be ruled out. A predictive KASP marker has also been developed and validated for efficient marker assisted selection, and an implementation strategy has been devised to ensure optimal gene stewardship.

## Methods

### Plant materials and pathogen isolates

We used the near‐isogenic line for *Rph15,* [BW719; PI 355447/7*Bowman – Martin *et al*., 2020a], as the resistance donor wild type for the development of mutants, population development and DNA template for complementation experiments. An F_2_‐F_3_ mapping population was developed by crossing BW719 with the leaf rust susceptible cv. Gus to re‐confirm linkage with the chromosome arm location and the inheritance of the *Rph15* resistance. Complementation analysis in barley was performed using barley cultivar Golden Promise. The donor accession for *Rph16* (HS‐680) was used for EMS mutation, detailed rust testing and for DNA extraction and Sanger sequencing. A detailed list of the rust pathogen isolates used in this study and their virulence phenotypes can be found in Table S2 and is also detailed further in Martin *et al*. (2020a).

### Candidate gene identification in Morex reference and amplification from Bowman + *Rph15*

Based on the hypersensitive infection type of the *Rph15* resistance, we used the physical interval of 44‐57Mb reported in Martin *et al*. (2020a) for the introgression of the *Rph15* resistance from PI 355447 into Bowman to search for predicted annotated NLR candidate resistance genes in the Morex reference assembly using the NLR annotator pipeline reported by Steuernagel *et al*. (2020). The sequence from 44 800 000 to 57 300 000 bp on chromosome 2H of the Morex reference genome (Mascher *et al*., 2017) was extracted by samtools version 1.9.0. The sequence was cut into fragments of 20 kb length with 5 kb overlap, and the NLR prediction was performed based as previously described (https://github.com/steuernb/NLR‐Annotator Steuernagel *et al*., 2020).

We extracted flanking sequences from the Morex reference around the candidate gene locus and design a single primer pair (RGA4 F: and RGA4 R) based on the Morex *rph15* candidate gene sequence (HORVU2Hr1G019120.5 GenBank accession AY641411.1) to amplify a 9442 base pair genomic fragment including approximately: 1.3 kb of sequence upstream from the translation Start codon and 2.5 kb of sequence downstream of the STOP codon. Polymerase chain reaction (PCR) was performed using Phusion polymerase as per the manufacturer’s instructions (New England Biosciences). PCR was performed using 50 µL reactions including: 1 × HF buffer, 100 ng of genomic DNA, 1 µm of primers, 3% of dimethyl sulfoxide (DMSO), 1 Unit of Phusion polymerase (New England Biosciences). PCR was performed at 1 cycle of 98°C for 3 min followed by 35 cycles of denaturing at 98°C for 20 s, annealing at 60°C for 20 s, extension at 72°C for 3 min and 30 s and then incubating at 72°C for 10 min. The PCR products were separated on a 1% agarose gel, and a single band of the expected size was excised and purified. The gel‐purified PCR product was then cloned into the sub‐cloning vector TOPO XL as per manufacturer’s instructions (Invitrogen). We amplified and cloned genomic fragments from resistant donors for *Rph15* (BW719) and *Rph16* (HS‐680) and the universally susceptible (Gus) barley accessions. The plasmid DNA of five positive clones from each amplicon was sent for Sanger sequencing using internal primers, and the sequences were compared with the template from Morex. All primers used in this study are detailed in Table S4.

### cDNA analysis

Total RNA was extracted using the TRIzol method (Invitrogen) as per manufacturer’s instructions. cDNA synthesis, 5′ and 3′ RACE (rapid amplification of cDNA ends) were performed using the manufacturer’s instructions (Clontech).

### Generation of loss of function mutants for *Rph15*

We performed sodium azide mutagenesis on the wild‐type *Rph15* donor barley line BW719 (Martin *et al*., 2020a,) using the procedure described by Chandler and Harding (2013) with some modifications. Approximately 1500 seeds were immersed in water at 4°C overnight. The imbibed seeds were transferred to a 2‐L measuring cylinder filled with water and aerated with pressurized air for 8 h, with one change of water after 4 h. The water was drained, and the seeds were incubated in a shaker for 2 h in freshly prepared 1 mm sodium azide dissolved in 0.1 m sodium citrate buffer (pH 3.0). Next, the seed was washed extensively in running water for at least 2 h and placed in a fume hood to dry overnight. Seeds were sown in the field, and single spikes from each plant were harvested separately from the remaining spikes, which were harvested in bulk.

The Bowman + *Rph15* mutant M_2_ spikes and M_2_‐derived M_3_ families were phenotypically assessed at the seedling stage as described by Dracatos *et al*. (2019). In all cases, at least two susceptible plants (putative *rph15* knockouts) were transplanted for each candidate segregating M_2_ family for subsequent progeny testing. Sequence confirmation for each mutant was performed through PCR amplification of M_3_ derived susceptible progeny for each mutant family. Mutagenesis was also previously performed on HS‐680 using the same approach, and susceptible knockouts were identified at the Julius Kühn‐Institute (JKI), Quedlinburg, Germany using the *P. hordei* isolate I‐80 (avirulent with respect to both *Rph15* and *Rph16*) (Table S2).

### Sequence confirmation of *rph15* mutants

Sanger sequence confirmation of all mutants was performed at the M_3_ stage using a three‐step process. Firstly, only DNA from progeny‐tested homozygous susceptible families was extracted using the CTAB method (Doyle and Doyle, 1987) for PCR amplification of the 9442 bp genomic fragment for the candidate *Rph15* gene as described above. Secondly, the PCR products were cloned into the TOPO XL vector as described above and three positive clones for each amplicon were sent for Sanger sequencing on a fee‐for‐service basis to the Australian Genome Research Facility (AGRF) for comparison with the wild‐type *Rph15* candidate gene. Finally, only when all three clones carried the same non‐synonymous sodium azide‐induced (either G to A or C to T) mutation were they deemed confirmed mutants.

### KASP marker development and validation for the *Rph15* resistance

A Kompetitive Allele‐Specific PCR (KASP) marker assay was designed by interrogating a C/G SNP within the 3^rd^ intron of the *Rph15* gene using the following primers: *Rph15* K3 FAM (resistant allele): 5′‐GAAGGTGACCAAGTTCATGCTGGGCTGTTATTAGCATGGTCCTC‐3′, *Rph15* K3 VIC (susceptible allele) : 5′‐GAAGGTCGGAGTCAACGGATTGGGCTGTTATTAGCATGGTCCTG‐3′, *Rph15* K3 R: 5‐AATACCACAATGACTACCCCAGGTT‐3′). The KASP assay was set using an 8 µL reaction volume, including 4 µL of KASP master mix (LGC), KASP Assay mix (0.12 pmol two allele‐specific primers and 0.3 pmol common reverse primer) and 25 to 50 ng of genomic DNA. The KASP marker reaction conditions were as follows: 1 cycle at 94°C for 15 min, 10 cycles of at 94°C for 20 s and 65°C for 1 min and then 32 cycles of at 94°C for 20 s and 57°C for 1 min, cooled down to 25°C for 5 min. Marker validation was performed using a panel of 80 Australian barley accessions all postulated to lack *Rph15* and 60 leaf rust resistant Bowman near‐isogenic lines of which many were postulated to carry the *Rph15* resistance. All accessions are listed in Table S1.

### Complementation using Agrobacterium‐mediated transformation (ABMT) in barley

The 9442 bp fragment containing its native promoter and terminator was amplified by PCR with *NotI* at both sites, enzyme cutting and ligation into pWBVec8 (Wang *et al*., 1998). Subsequently, the vector containing the intact sequence‐verified *Rph15* candidate gene was transformed into *Agrobacterium tumefaciens* strain AgL0 using the freeze/thaw method (Chen *et al*., 1994). Barley transformation into Golden Promise was performed as described by Bartlett *et al*. (2008).

### Quantitative expression analysis of *Rph15*

The relative expression of the *Rph15* resistance gene was assessed in two different mutant lines and the Golden Promise + *Rph15* transgenic lines including the same four T_1_ transgenic lines that were also rust tested against the *Rph15*‐virulent *P. hordei* isolate 90‐3 from Israel. Barley genotypes Bowman, BW719, mutant lines M4022 and M1727 were grown in the glasshouse for 12 days at 20°C. Ten‐day‐old seedlings were inoculated with *P. hordei* pathotype 5457 P+, and both uninfected and infected leaf samples were harvested at two time‐points (0 and 24 h after inoculation, respectively). For the transgenics, DNA was extracted from six half seeds for each of the Golden Promise + *Rph15* T1 transgenic lines (B49‐1, B49‐2, B49‐3, B49‐8a, B49‐8c, B49‐11a and B49‐11c) and subsequently genotyped with the *Rph15* KASP marker. Marker positive transgenic resistant sib lines, Bowman + *Rph15*, Golden Promise and Bowman were grown in the growth chamber, and tissue was sampled at the 2nd‐3rd leaf stage for RNA extraction. RNA was extracted using Plant RNA Kit (Promega) on Maxwell® RSC 48 Instrument (Promega) as per manufacturer’s instructions. cDNA was synthesized from 1 µg of total RNA using SuperScript™ III Reverse Transcriptase (Invitrogen) as per manufacturers instructions. The total volume was brought to 100 µL with water after the reaction was finished.

qPCR was performed by adding 5 µL of iTaq Universal SYBR® Green Supermix (BIO‐RAD), 1 µL of forward primer (10 µm), 1 µL of reverse primer (10 µm) and 3 µL of diluted cDNA, using the standard protocol. PCR reactions were performed at 1 cycle of 95°C for 3 min, 40 cycles of denaturing at 95°C for 10 s, annealing (extension) at 60°C for 30 s with plate reading at each cycle and melt curve from 65°C to 95°C with increment of 0.5°C for 5 s plus plate reading. Reactions for each cDNA sample contain two sets of primers: 1/reference gene primers Hv‐ACT‐F and Hv‐ACT‐R (*Hordeum vulgare* actin gene) and 2/*Rph15* specific primers Rph15_1679‐F and Rph15_1793‐R (Table S5). The relative expression level is expressed as: RE = 2^ΔCt^ where ΔCt = Ct(*actin*) – Ct(*Rph15*). The relative expression levels of all samples were normalized as the relative expression of Bowman + *Rph15* was set to 100.

### Nearest neighbour analysis using the *Hordeum* exome capture

The genomic sequence of *Rph15* was aligned to the genome sequence assembly of barley cultivar Morex (Morex V1, Mascher *et al*., 2017) using the BWA‐MEM algorithm (Li 2013) set to default parameters. Sequence variants between Morex and *Rph15* were detected with BCFtools mpileup (Li 2011). The results were imported into R (R Core Team, 2016) and intersected with a SNP matrix derived from exome capture data of 267 wild and domesticated barley accessions (Russell *et al*., 2016). Nearest neighbours of *Rph15* in a set of 91 wild barleys were found by a simple SNP matching distance. A map showing the collection sites of the 55 neighbours was plotted using the R package mapdata (https://cran.r‐project.org/web/packages/mapdata/index.html) (Figure 5). Collection sites were taken from Russell *et al*. (2016). The collection site of the *Rph15*‐virulent isolate (90‐3) in Sha’ar HaGai, Israel was superimposed on the same map to illustrate the colocation between the putative origin of the *Rph15* resistance and matching virulence within *P. hordei* populations, respectively.

### Phylogenetic analysis

The predicted *Rph15* amino acid sequence (HsRph15) was used as a query in GenBank using the program BlastP to identify closely related sequences. The HsRph15 amino acid sequence was then compared with that of related NLR sequences from *Oryza sativa* (Os) Xa1 (BAM17617.1) Triticeae including: *Aegilops tauschii* (Ata) (Sr33‐AGQ17384.1, Sr45‐CUM44213.1 and Lr22a‐ARO38244.1), *Secale cerale* (Sc) (Sr50‐ALO61074.1 and Pm8‐AGY30894.1), *Triticum aestivum* (Ta) (TaYr5, TaYr7, Pm2‐CZT14023.1), *T. monococcum* (Tm) (Sr22‐CUM44212.1, Sr35‐AGP75918.1 and MLA1‐ADX06722.1), *T. dicoccoides* (Td; Lr10‐ADM65840.1) and *Hordeum vulgare* (Hv; rph15 v2, Rph1‐MK376319, MLA1‐AAG37354.1, MLA6‐CAC29242.1, and MLA9‐ACZ65487.1). An unrelated NLR from Arabidopsis (*Arabidopsis thaliana*), At5g45510‐Q8VZC7.2, was included as an out‐group. A multiple sequence alignment was performed using ClustalW (Larkin *et al*., 2007), in Geneious version 11.0.2 (https://www.geneious.com) with the BLOSUM scoring matrix and settings of gap creation at 210 cost and gap extension at 20.1 cost per element. After removing all ambiguously aligned regions using trimAl (Capella‐Gutiérrez *et al*., 2009), the final sequence alignment of length 1826 amino acids (*n* = 18) was determined. The BED domains of Xa1, Rph15, Yr5 and Yr7 were removed, and a 2nd multiple sequence alignment was performed and a phylogram was constructed (BED‐). Two phylogenetic trees were constructed based on BED+ and BED‐ multiple sequence alignments. Both alignments were then inferred using the neighbour‐joining method in the Geneious Tree Builder software, employing the Jukes‐Cantor genetic distance model. Bootstrap support for individual nodes was generated using 1,000 bootstrap replicates.

## Competing interests

The authors declare no competing financial interests.

## Author contribution

C.C., P.M.D., B.C., O.M., Martin, M., T.R. and S.P. performed experiments. P.M.D., R.F.P, B.J.S., D.S., D.P. and J.F. developed and provided essential biological material. Mascher M and M.J. performed bioinformatics. R.F.P and E.S.L provided infrastructure and funding for the project. P.M.D. wrote the manuscript with contribution from C.C. All authors approved the final manuscript before submission.

## Supporting information


**Figure S1** Confirmation of the BW719 genetic stock as the wild type source of *Rph15* used in this study.

**Figure S2** Leaf rust infection phenotypes 11 days after inoculation of (L to R) wild type BW719 (Bowman+*Rph15*), Bowman, and the six sodium azide‐induced non‐synonymous *rph15* knockout mutants: M1371‐3, M1695, M1727, M4022, M4321 and M4651.

**Figure S3** Summary of phenotypic and molecular characterisation of the T_0_ generation Golden Promise+*Rph15* and control lines.

**Figure S4** Phenotypic assessment of seedlings from (L to R) Bowman+*Rph15* (BW719), Golden Promise and individual sib plants from T_1_ generation Golden Promise+*Rph15* transgenic lines B49‐2 and B49‐11a inoculated with North American *Puccinia hordei* races that are virulent (90‐3, A) and avirulent (92‐7, B) with respect to *Rph15*. For a full description of infection types refer to Park et al. (2015).

**Figure S5** Relative expression analysis using qRT‐PCR of the *Rph15* resistance gene in the different T_1_ generation Golden Promise+*Rph15* transgenic lines generated in this study relative to the resistant wild type Bowman+*Rph15* (BW719) and susceptible genotypes Golden Promise (GP) and Bowman (Bo).

**Figure S6** Comparative amino acid sequence alignment of the predicted BED domains of the NLR proteins from Rph15 from BW719 identified in this study with Yr5 and Yr7 from bread wheat (Marchal et al. 2018) and Xa1 from rice (GenBank accession BAA25068.1).

**Figure S7** The geographic distribution of 91 wild barleys (*Hordeum* *vulgare* ssp. *spontaneum*) from the exome capture based on collection sites from the Fertile Crescent as given by Russell et al. (2016).

**Table S1** Genotypic results of 61 near‐isogenic lines carrying leaf rust resistance in cultivar Bowman published in Martin et al. (2020) with the gene‐based *Rph15* KASP SNP marker developed in this study.

**Table S2** Summary table of leaf rust pathogen isolates used in this study and their virulence/avirulence spectra.

**Table S3** Summary of phenotypic data of four T_1_ families and barley leaf rust differential genotypes infected with two *Puccinia hordei* isolates with contrasting virulence for *Rph15*.

**Table S4** Phenotypic response testing at the seedling stage of barley leaf rust differential host genotypes at the University of Minnesota, US using six pathogenically diverse *Puccinia hordei* isolates. The locations and details of these isolates are specified in Martin et al. (2020).

**Table S5** Summary table of the primers used in this study.
