# Supplementary figures and images for: BED domain‐containing NLR from wild barley confers resistance to leaf rust
Source: Plant Biotechnol J. 2021 Mar 6;19(6):1206–15. doi: 10.1111/pbi.13542 (PMC8196641; doi:10.1111/pbi.13542)

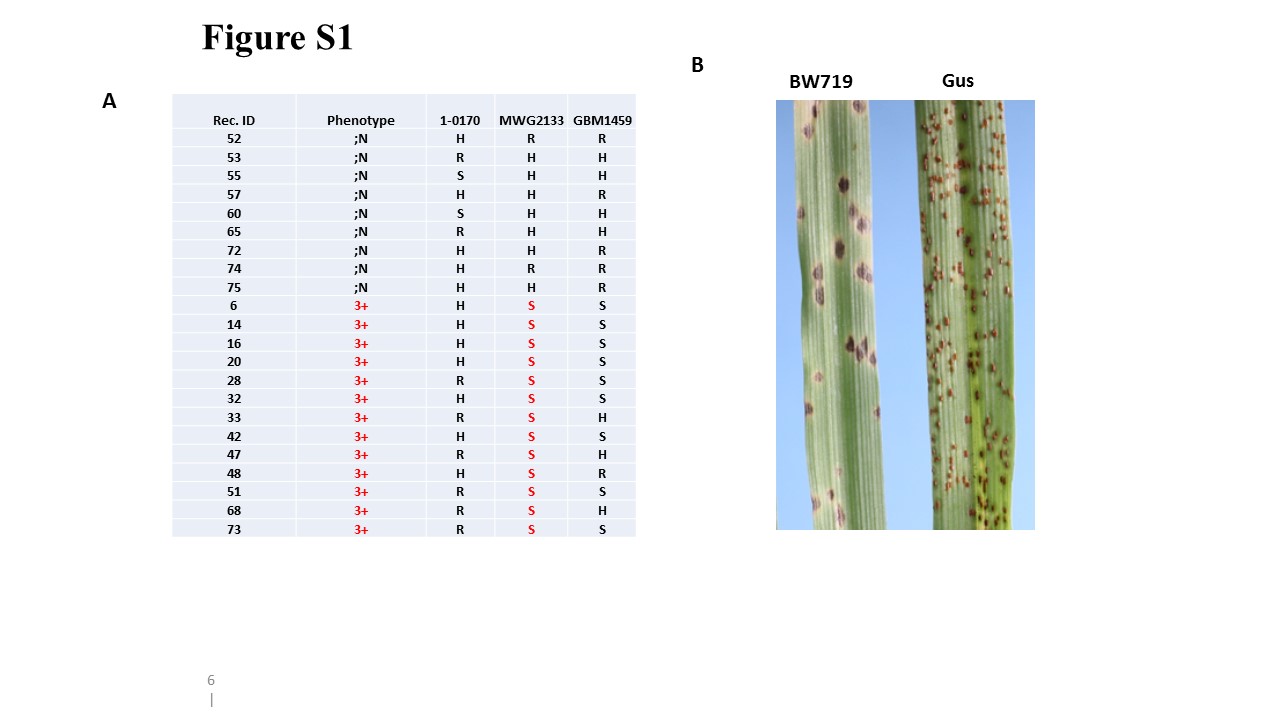

Supplement: Supplementary file 1 — Figure S1 Confirmation of the BW719 genetic stock as the wild type source of Rph15 used in this study. [file PBI-19-1206-s008.jpg]

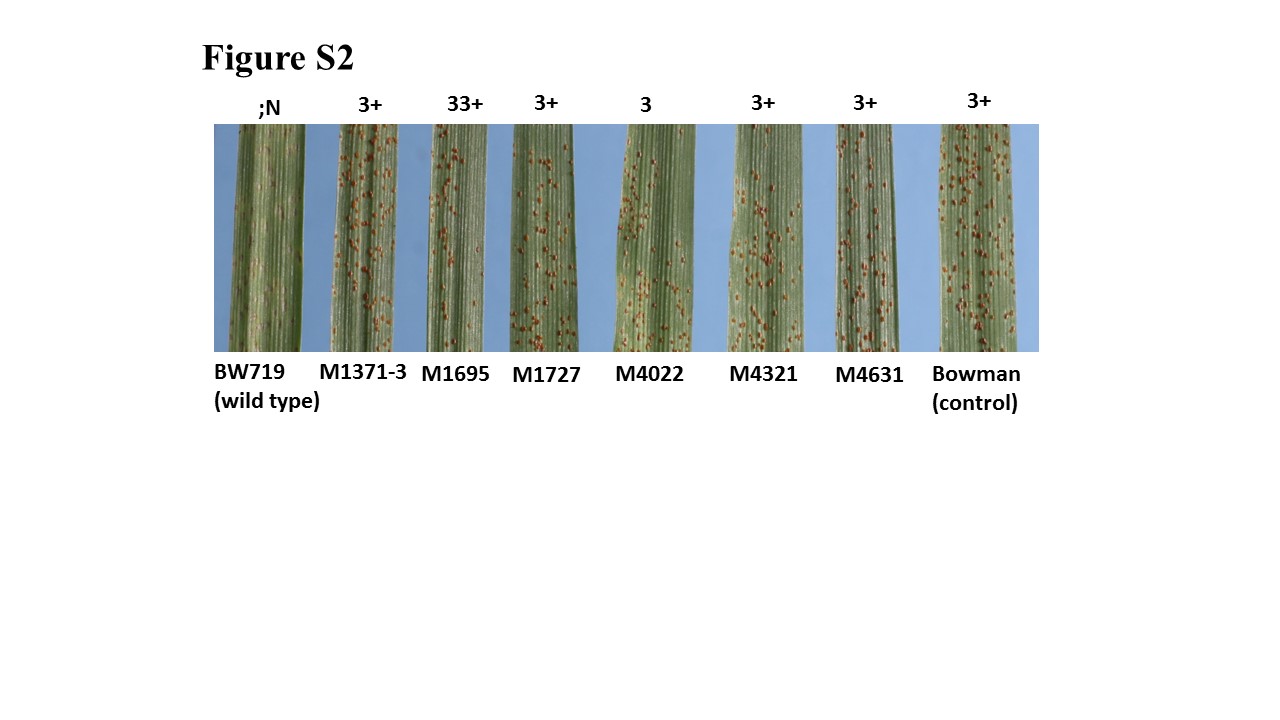

Supplement: Supplementary file 2 — Figure S2 Leaf rust infection phenotypes 11 days after inoculation of (L to R) wild type BW719 (Bowman+Rph15), Bowman, and the six sodium azide‐induced non‐synonymous rph15 knockout mutants: M1371‐3, M1695, M1727, M4022, M4321 and M4651. [file PBI-19-1206-s005.jpg]

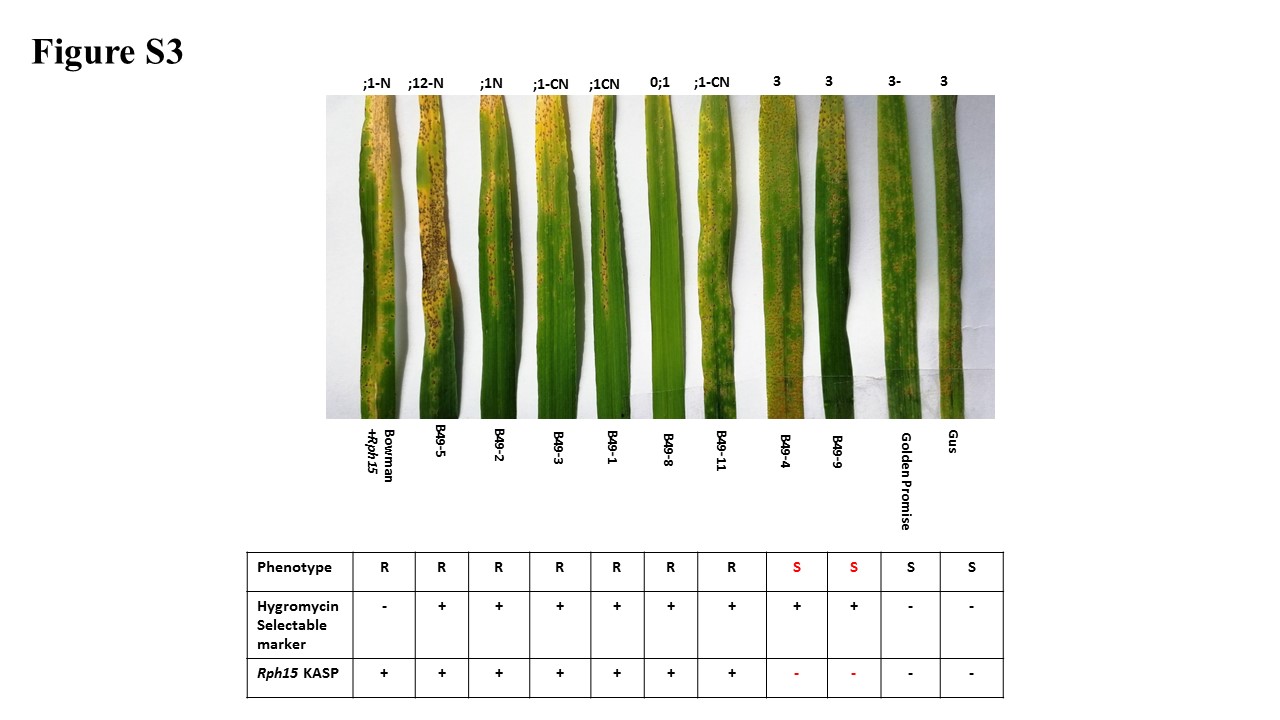

Supplement: Supplementary file 3 — Figure S3 Summary of phenotypic and molecular characterisation of the T0 generation Golden Promise+Rph15 and control lines. [file PBI-19-1206-s007.jpg]

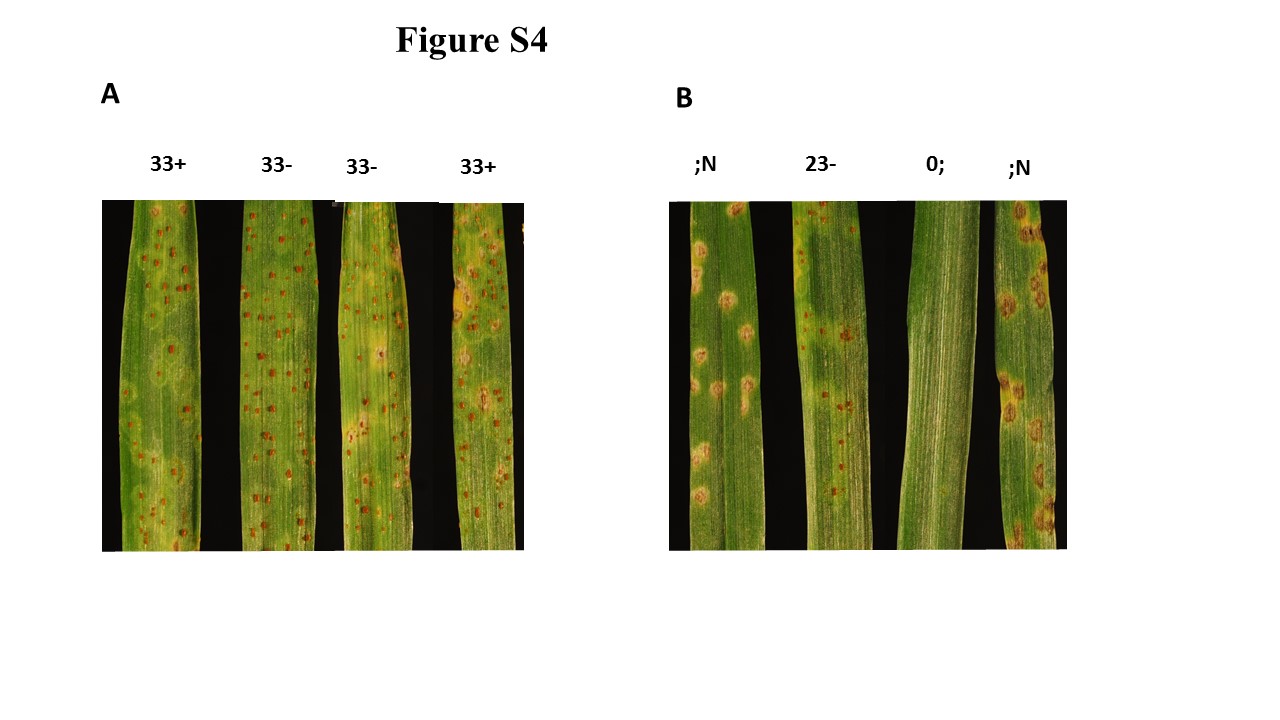

Supplement: Supplementary file 4 — Figure S4 Phenotypic assessment of seedlings from (L to R) Bowman+Rph15 (BW719), Golden Promise and individual sib plants from T1 generation Golden Promise+Rph15 transgenic lines B49‐2 and B49‐11a inoculated with North American Puccinia hordei races that are virulent (90‐3, A) and avirulent (92‐7, B) with respect to Rph15. For a full description of infection types refer to Park et al. (2015). [file PBI-19-1206-s001.jpg]

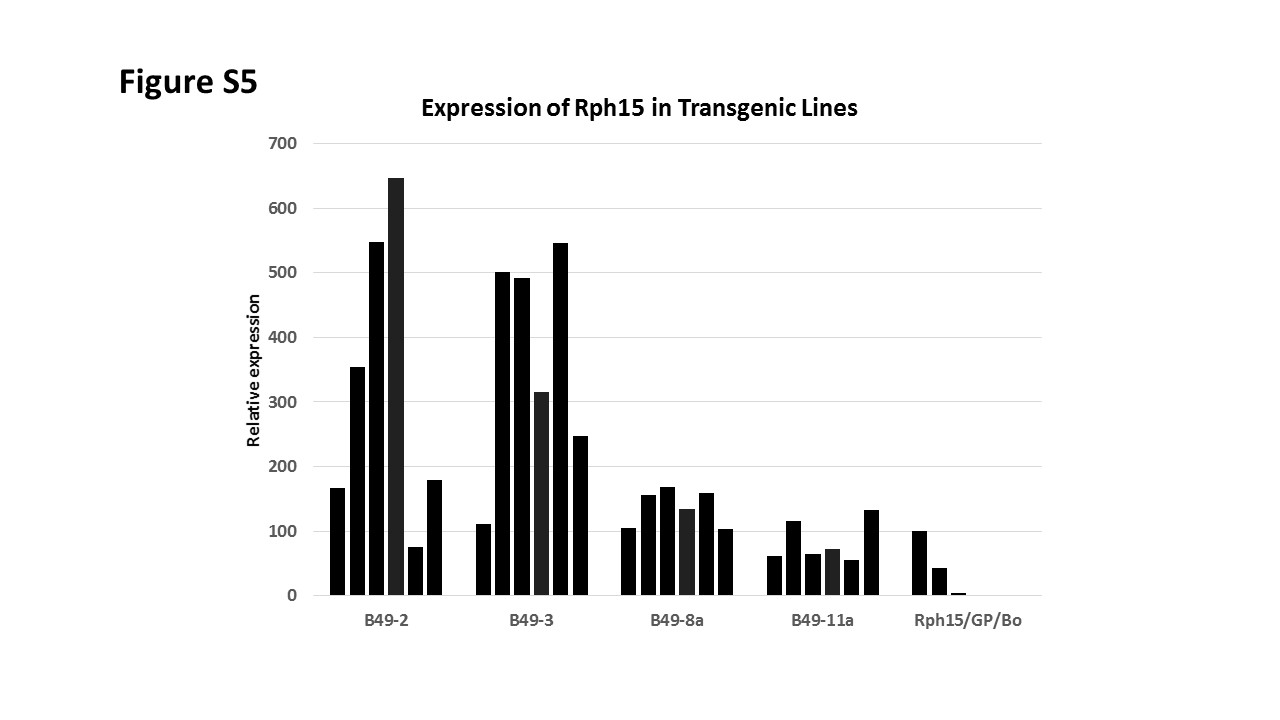

Supplement: Supplementary file 5 — Figure S5 Relative expression analysis using qRT‐PCR of the Rph15 resistance gene in the different T1 generation Golden Promise+Rph15 transgenic lines generated in this study relative to the resistant wild type Bowman+Rph15 (BW719) and susceptible genotypes Golden Promise (GP) and Bowman (Bo). [file PBI-19-1206-s011.jpg]

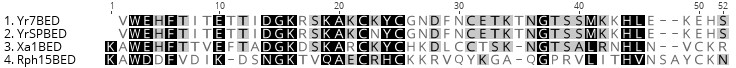

Supplement: Supplementary file 6 — Figure S6 Comparative amino acid sequence alignment of the predicted BED domains of the NLR proteins from Rph15 from BW719 identified in this study with Yr5 and Yr7 from bread wheat (Marchal et al. 2018) and Xa1 from rice (GenBank accession BAA25068.1). [file PBI-19-1206-s002.jpg]

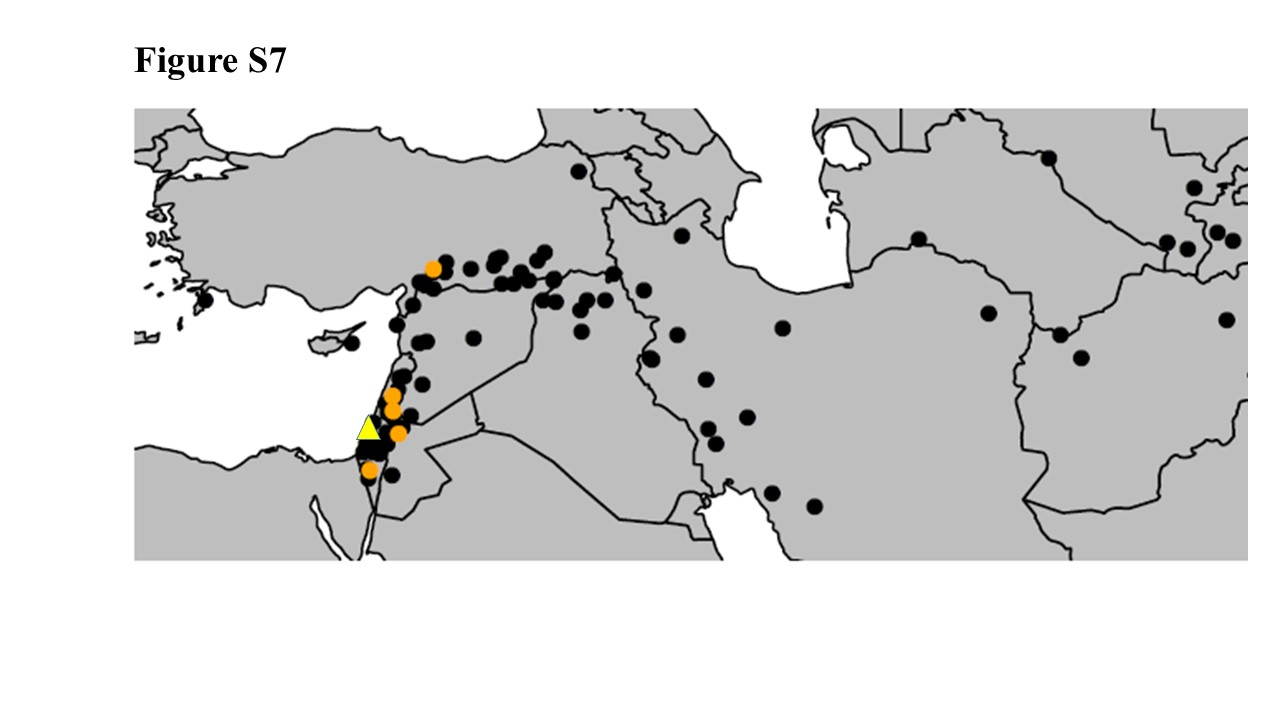

Supplement: Supplementary file 7 — Figure S7 The geographic distribution of 91 wild barleys (Hordeum vulgare ssp. spontaneum) from the exome capture based on collection sites from the Fertile Crescent as given by Russell et al. (2016). [file PBI-19-1206-s012.jpg]
