# Supplementary material for: BED domain‐containing NLR from wild barley confers resistance to leaf rust
Source: Plant Biotechnol J. 2021 Mar 6;19(6):1206–15. doi: 10.1111/pbi.13542 (PMC8196641; doi:10.1111/pbi.13542)
Supplement: Supplementary file 8 — Table S1 Genotypic results of 61 near‐isogenic lines carrying leaf rust resistance in cultivar Bowman published in Martin et al. (2020) with the gene‐based Rph15 KASP SNP marker developed in this study. [file PBI-19-1206-s009.docx]

**Supplementary Table 1**- Genotypic results of 61 near isogenic lines carrying leaf rust resistance in the Bowman background published in Martin et al. (2020) with the gene-based *Rph15* KASP SNP marker developed in this study.

| **Line** | **Allele** | **Pedigree** | **KASP marker allele** |
| --- | --- | --- | --- |
| BW667 | *Rph15* | PI405338/3*Bowman | *Rph15* |
| BW668 | *?* | Bowman*4/PI405169A | allele other than Bowman and *Rph15* |
| BW669 | *Rph15* | PI405298/4*Bowman | *Rph15* |
| BW670 | *Rph15* | PI391007/4*Bowman | *Rph15* |
| BW671 | *Rph15* | PI466470/5*Bowman | *Rph15* |
| BW672 | *?* | Bowman*5//Moore/PI466373 | allele other than Bowman and *Rph15* |
| BW673 | *Rph15* | Bowman*6//Aim/PI405303 | *Rph15* |
| BW674 | *Rph15* | Bowman*7/PI466245 | *Rph15* |
| BW678 | *Rph15* | Bowman*4/PI354926 | *Rph15* |
| BW679 | *Rph15* | Bowman*6/PI282610 | *Rph15* |
| BW680 | *Rph15* | PI391049/4*Bowman | *Rph15* |
| BW681 | *Rph15* | PI391044/ND13944//4*Bowman | *Rph15* |
| BW688 | *Rph15* | PI354940/5*Bowman | *Rph15* |
| BW689 | *Rph15* | Bowman*5/PI405277 | *Rph15* |
| BW690 | *Rph15* | PI354923/5*Bowman | *Rph15* |
| BW691 | *Rph15+?* | Bowman*5/PI391045A het Eam1 | allele other than Bowman and *Rph15* |
| BW692 | *Rph15* | PI296841/6*Bowman | *Rph15* |
| BW693 | *Rph15* | PI354932/6*Bowman | *Rph15* |
| BW694 | *Rph15* | PI391002/6*Bowman | *Rph15* |
| BW695 | *Rph15* | PI391072/6*Bowman | *Rph15* |
| BW696 | *Rph15* | PI391087/6*Bowman | *Rph15* |
| BW697 | *Rph15+?* | Bowman*6/PI391121 | allele other than Bowman and *Rph15* |
| BW698 | *?* | Bowman*6/PI405154 | Bowman |
| BW699 | *Rph15* | PI405194/6*Bowman | *Rph15* |
| BW700 | *Rph15* | Bowman*6/PI405201 | *Rph15* |
| BW701 | *?* | PI405215/ND13944//5*Bowman | allele other than Bowman and *Rph15* |
| BW702 | *Rph15* | Bowman*6/PI405219 | *Rph15* |
| BW703 | *Rph15* | PI405233/6*Bowman | *Rph15* |
| BW704 | *Rph15* | PI405289/6*Bowman | *Rph15* |
| BW705 | *Rph15* | PI405308/6*Bowman | *Rph15* |
| BW706 | *Rph15* | Bowman*6/PI405332 | *Rph15* |
| BW707 | *Rph15* | Bowman*6/PI405335 | *Rph15* |
| BW708 | *Rph15* | Bowman*6/PI405364 | allele other than Bowman and *Rph15* |
| BW709 | *Rph15+?* | Bowman*6/PI405399 | *Rph15* |
| BW710 | *Rph15* | Bowman*7/PI405235 | *Rph15* |
| BW711 | *Rph15* | Bowman*7/PI405311 | *Rph15* |
| BW712 | *Rph15* | PI354928/4*Bowman | allele other than Bowman and *Rph15* |
| BW713 | *Rph15* | PI391000/6*Bowman | *Rph15* |
| BW714 | *Rph15* | PI391024/6*Bowman | *Rph15* |
| BW715 | *Rph15* | Bowman*6/PI355444 | *Rph15* |
| BW716 | *Rph15* | Bowman*6/PI405227 | allele other than Bowman and *Rph15* |
| BW717 | *Rph15* | PI405236/6*Bowman | *Rph15* |
| BW718 | *Rph15* | Bowman*6/PI405354A | *Rph15* |
| BW719* | *Rph15* | PI 355447/8*Bowman | *Rph15* |
| BW720 | *Rph15* | PI391089/8*Bowman | *Rph15* |
| BW721 | *Rph15* | Bowman*8/PI354937 | *Rph15* |
| BW722 | *Rph15* | Bowman*8/PI391069 | *Rph15* |
| BW723 | *Rph15* | PI405179/4*Bowman 0:cn | *Rph15* |
| BW724 | *Rph15* | Bowman*6/PI405341 | *Rph15* |
| BW725 | *Rph15* | PI391004/7*Bowman Translocation | *Rph15* |
| BW726 | *?* | HS 584/Bowman | *Rph15* |
| BW727 | *?* | HS 580/Bowman | Bowman |
| BW728 | *?* | Bowman*4/PI355445 | allele other than Bowman and *Rph15* |
| BW729 | *Rph15* | Bowman*5/PI355434 | *Rph15* |
| BW730 | *Rph15* | PI405210/5*Bowman | *Rph15* |
| BW733 | *Rph15* | PI405220/6*Bowman | allele other than Bowman and *Rph15* |
| BW734 | *Rph15* | PI466483/6*Bowman | *Rph15* |
| BW735 | *?* | 81882 *Rph17*.af/Bowman | Bowman |
| BW736 | *?* | Bowman*2/38P18-9-4 | Bowman |
| BW749 | *?* | Bowman*6/TUNISIA 33 | allele other than Bowman and *Rph15* |
| BW751 | *?* | 12022/3/CMB822/BOW//Columbia/4/6*Bowman | allele other than Bowman and *Rph15* |
| Controls |  | Bowman+*Rph15* (Park differential) | *Rph15* |
| Controls |  | Bowman+*Rph15* (Park differential) | *Rph15* |
| Controls |  | Bowman | Bowman |
| Controls |  | Bowman | Bowman |
| Controls |  | Bowman+*Rph15* (Park differential) plus Bowman | heterozygous call |
