# Supplementary material for: BED domain‐containing NLR from wild barley confers resistance to leaf rust
Source: Plant Biotechnol J. 2021 Mar 6;19(6):1206–15. doi: 10.1111/pbi.13542 (PMC8196641; doi:10.1111/pbi.13542)
Supplement: Supplementary file 9 — Table S2 Summary table of leaf rust pathogen isolates used in this study and their virulence/avirulence spectra. [file PBI-19-1206-s010.docx]

**Table S2** - Summary table of leaf rust pathogen isolates used in this study and their virulence/avirulence spectra

|  |  | |
| --- | --- | --- |
| **Pathotype name** | | **avirulence/virulence spectra** |
| 5457 P+ | | *Rph5,* 7,13,15,17,18, 20, 21/1, 2, 3, 4, 6, 8, 9,12,19 |
| 90-3 | | *Rph3,* 5, 7, 10, 11, 1, 17, 18/1, 2, 4, 6, 8, 9, 12, 14, 15, 16, 19 |
| 92-7 | | *Rph11,* 13, 14, 15, 17, 18, 20/1, 2, 3, 4, 5, 6, 7, 8, 9, 10, 12, 19 |
| 89-3 | | *Rph3,* 13, 14, 15, 16/1, 2, 4, 5, 7, 8, 9, 10, 11, 12 |
| Neth28 | | *Rph3,* 4, 9, 13, 15, 16/1, 2, 5, 6, 7, 8, 10, 11, 12, 14 |
| 92-6 | | *Rph9,* 12, 13, 14, 15, 16/1, 2, 3, 4, 5, 6, 7, 8, 10, 11 |
| 90-5 | | *Rph3,* 5, 7, 15, 16/1, 2, 4, 6, 8, 9, 10, 11, 12, 13, 14 |
| I-80 | | *Rph7*,13,15,16/1, 2, 3, 4, 5, 6, 8, 9, 12, 14 |
