# Supplementary material for: BED domain‐containing NLR from wild barley confers resistance to leaf rust
Source: Plant Biotechnol J. 2021 Mar 6;19(6):1206–15. doi: 10.1111/pbi.13542 (PMC8196641; doi:10.1111/pbi.13542)
Supplement: Supplementary file 11 — Table S4 Phenotypic response testing at the seedling stage of barley leaf rust differential host genotypes at the University of Minnesota, US using six pathogenically diverse Puccinia hordei isolates. The locations and details of these isolates are specified in Martin et al. (2020). [file PBI-19-1206-s006.docx]

**Table S4** Phenotypic response testing at the seedling stage of barley leaf rust differential host genotypes at the University of Minnesota, US using six pathogenically diverse *Puccinia hordei* isolates. The locations and details of these isolates are specified in Martin et al. (2020).

|  |  |  |  |  |  |  |
| --- | --- | --- | --- | --- | --- | --- |
|  |  |  |  |  |  |  |
|  |  |  |  |  |  |  |
| **Differential host genotype** | **89-3** | **Neth28** | **90-3** | **92-7** | **92-6** | **90-5** |
| Sudan (*Rph1*) | S | S | S | S | S | S |
| Peruvian (*Rph2*) | S | S | S | S | S | S |
| Estate (*Rph*3) | R | R | R | S | S | R |
| Gold (*Rph4*) | S | R | S | S | S | S |
| Magnificent (*Rph5*) | S | S | S | S | S | R |
| Bowman/Bol (*Rph6*) | S | S | S | S | S | S |
| Cebada capa (*Rph7*) | S | S | R | S | S | R |
| Egypt 4 (*Rph8*) | S | S | S | S | S | S |
| Hor2596 (*Rph9*) | S | R | S | S | R | S |
| Clipper BC8 (*Rph10*) | S | S | S | S | S | S |
| Clipper BC67 (*Rph11*) | S | S | S | S | S | S |
| Triumph (*Rph12*) | S | S | S | S | R | S |
| PI 531849 (*Rph13*) | R | R | R | R | R | S |
| PI 584760 (*Rph14*) | R | S | S | S | R | S |
| I95-282-2 (*Rph15*) | **R^a^** | **R** | **S^b^** | **R** | **R** | **R** |
| HS084 (*Rph16*) | **R** | **R** | **S** | **R** | **R** | **R** |

^a^ Resistant and ^b^ Susceptible infection responses are highlighted yellow and red to denote isolate specificity between differential stocks carrying *Rph15* and *Rph16* using the 6 diverse *P. hordei* isolates.
