# Supplementary material for: BED domain‐containing NLR from wild barley confers resistance to leaf rust
Source: Plant Biotechnol J. 2021 Mar 6;19(6):1206–15. doi: 10.1111/pbi.13542 (PMC8196641; doi:10.1111/pbi.13542)
Supplement: Supplementary file 12 — Table S5 Summary table of the primers used in this study. [file PBI-19-1206-s003.docx]

**Supplementary Table 5. Summary table of the primers used in this study**

| **Primer Name** | **Squence(5'→3')** | **Tm** | **Application** |
| --- | --- | --- | --- |
| RGA4 F1 | CATTATCATAAGTGTAACCCCTGCAG | 60.7 | cloning of Rph15 |
| RGA4 R1 | TGCCGGATTATTTGCTCAGA | 60.1 | cloning of Rph15 |
| RGA4 F2 | CCCTTTGCTTGTGGGGACT | 60.9 | sequencing |
| RGA4 F3 | CATGGGATGATTTTGTCGACA | 60.2 | sequencing |
| RGA4 F4 | TGGGTGTCTAACAGCTTGGATG | 60.9 | sequencing |
| RGA4 F5 | TCTGGTCTAATGCATGATTTTGC | 59.9 | sequencing |
| RGA4 F6 | CTGAAAGTTCTAGCAGCGGTGT | 59.5 | sequencing |
| RGA4 F7 | AGAGCATGCCCTCCCATC | 59.5 | sequencing |
| RGA4 F8 new | ATTGAAATCCCTACAGCTGCACT | 60.0 | sequencing |
| RGA4 F9 | TCCACCATCAGTTTACGGTAAAAG | 60.1 | sequencing |
| RGA4 F10 | CGAAGAGATCAGAAGGTACGAGG | 60.2 | sequencing |
| XL-TOPO R | CACAGGAAACAGCTATGACCATG | 60.4 | sequencing |
| XL-TOPO F | AGGGTTTTCCCAGTCACGAC | 59.8 | sequencing |
| RGA4 F2 ATG | ATGGAGGACGCTTACCTTGTG | 60.0 | Mutant confirmation |
| Rph15 R-STOP | TCAGTGCACATATTGGTGGTCA | 60.8 | Mutant confirmation |
| Rph15 3'RACE -2 | TCAGGAAGTTGGGACTCTACAACAACCAGG | 70.1 | Rph15 RACE |
| Rph15 3'RACE-2 nest | TGTTACTGGAAGAATTGGATATTCGGGGC | 69.4 | Rph15 RACE |
| Rph15 5'RACE-2 | GTCCGCGAGAGATTTATCCAGCCGCGC | 76.7 | Rph15 RACE |
| Rph15 5'RACE-2 nest | ACCCCCTTTGCATCGGAAGCCAG | 71.6 | Rph15 RACE |
| RGA4 F1 Not | AAAGCGGCCGCCATTATCATAAGTGTAACCCCTGCAG | 60.7 | Cloning to binary vector |
| RGA4 R1 Not | AAAGCGGCCGCCTGCCGGATTATTTGCTCAGA | 60.1 | Cloning to binary vector |
| Hv-ACT-F | CGACAATGGAACCGGAATG | 66.6 | qPCR |
| Hv-ACT-R | CCCTTGGCGCATCATCTC | 67.3 | qPCR |
| Rph15_1679-F | GTAGTGGACGCATCATGTCTTCTT | 65.6 | qPCR |
| Rph15_1793-R | AGCAAGAAACTCATCATAGTCGGA | 65.2 | qPCR |
| Rph15 K3 FAM (resistant allele) | GAAGGTGACCAAGTTCATGCTGGGCTGTTATTAGCATGGTCCTC | 66.8 | KASP |
| Rph15 K3 VIC (susceptible allele) | GAAGGTCGGAGTCAACGGATTGGGCTGTTATTAGCATGGTCCTG | 67.6 | KASP |
| Rph15 K3 R | AATACCACAATGACTACCCCAGGTT | 66.4 | KASP |
